# Beyond biomarker validation: defining when serum neurofilament light chain should influence management in multiple sclerosis

**DOI:** 10.3389/fimmu.2026.1979820

**Published:** 2026-10-06

**Authors:** Ana Belén Caminero

**Affiliations:** Department of Neurology, Complejo Asistencial de Ávila, Ávila, Spain

**Keywords:** biomarkers, clinical decision-making, clinical utility, disease monitoring, multiple sclerosis, neuroimmunology, personalized medicine, serum neurofilament light chain

## Abstract

Serum neurofilament light chain (sNfL) is a well-established biomarker of neuroaxonal injury in multiple sclerosis (MS), associated with inflammatory disease activity, treatment response, and future clinical outcomes. As a downstream marker of tissue injury rather than a direct readout of a specific immune pathway, its increasing availability raises a more demanding question: when does sNfL provide enough additional information to improve management of an individual patient? This review examines sNfL from the perspective of clinical actionability and personalized decision support rather than biomarker validity alone. Reliable patient-level use requires analytical comparability, consideration of biological and clinical confounders, individualized normalization, temporal context, and longitudinal interpretation. Even when these conditions are met, clinical value depends on whether sNfL adds meaningful information beyond established clinical and magnetic resonance imaging monitoring. Current evidence best supports its role as a decision-support biomarker: unexpected or persistent abnormalities may justify verification, closer surveillance, or targeted reassessment, whereas normal values cannot reliably exclude clinically relevant MS biology, including relapse-independent disability accumulation. This asymmetry, together with the limited disease specificity of sNfL, argues against universal treatment thresholds and supports complementary biomarker and multimodal approaches. Evidence that sNfL predicts outcomes or influences intended management does not establish that acting on this information improves patient outcomes. Prospective studies are beginning to address this translational gap by evaluating sNfL-informed management strategies. The key remaining step is to demonstrate that acting on sNfL information—not merely measuring or interpreting it—improves clinically meaningful outcomes. Until then, sNfL is best positioned for biomarker-informed personalized monitoring rather than fully biomarker-guided care.

## 1 Introduction

Serum neurofilament light chain (sNfL) has become one of the most extensively studied blood biomarkers in multiple sclerosis (MS). A large body of evidence supports its association with inflammatory disease activity, magnetic resonance imaging (MRI) abnormalities, treatment response, and future clinical outcomes. The development of ultrasensitive assays and, more recently, age- and body mass index (BMI)-adjusted reference values has further increased its potential for use at the individual-patient level (1–3). Consequently, the question surrounding sNfL is progressively shifting from whether it is a biologically and clinically informative biomarker to how its information should be used in clinical practice.

In an immune-mediated disease, the biological position of sNfL is important. It is a downstream marker of neuroaxonal injury, not a direct measure of a specific immune pathway. Acute focal inflammation and inflammatory demyelination are major drivers of sNfL release in relapsing MS, and effective disease-modifying therapies generally reduce this signal by suppressing inflammatory injury. However, the same circulating signal can arise from non-inflammatory or non-MS causes of axonal damage and does not identify the cellular mechanism responsible for injury or select a particular immunotherapy. This combination of biological relevance and etiological non-specificity makes sNfL highly pertinent to neuroimmunology, but also explains why it cannot function as an autonomous treatment trigger (2, 3).

This transition raises a more difficult question. MS monitoring already integrates relapses, neurological examination, MRI findings, treatment history, and patient characteristics. The relevant issue is therefore not simply whether sNfL is associated with disease activity or prognosis, but whether it provides information that is sufficiently reliable and incremental to change the assessment or management of an individual patient (4, 5). Its clinical meaning depends not only on the measured concentration, but also on the context in which that information is obtained and on what was already known before the result became available.

Individual-level interpretation adds further complexity. sNfL is sensitive to neuroaxonal injury but is not specific to MS, and its circulating concentration is influenced by patient-related, analytical, and temporal factors. Moreover, an isolated concentration and a longitudinal change within the same patient may provide substantially different information (1–3). These considerations challenge the use of universal thresholds and favor individualized and longitudinal approaches to interpretation.

However, better interpretation does not by itself establish clinical actionability. This issue is increasingly relevant as sNfL measurement becomes more accessible and expert recommendations address its clinical implementation (2). Evidence that disclosure of sNfL results can influence clinical decisions and increase physicians’ certainty is beginning to emerge (6), while prospective studies are investigating sNfL-informed monitoring, implementation, and management strategies (7, 8). However, evidence that these strategies improve patient-relevant outcomes compared with otherwise comparable conventional care remains insufficient. Rather than providing another comprehensive review of the biological validity, prognostic associations, or treatment responsiveness of sNfL, this review focuses specifically on the translational gap between obtaining an sNfL result and knowing what to do with it in an individual patient.

A recent implementation-focused narrative review summarized biological interpretation, longitudinal use, confounders, and practical integration of sNfL with clinical assessment, MRI, and complementary biomarkers across the MS continuum (9). The present review does not repeat that broad implementation map. It addresses a narrower methodological question: what successive evidence is required before an interpretable sNfL result can be considered actionable and used to influence management in an individual patient?. Specifically, rather than focusing on how sNfL should be measured and interpreted in routine care, the present review examines the evidentiary transitions from association to individual discrimination, incremental value, decision impact, and ultimately outcome benefit. These distinctions were not the organizing framework of the previous implementation-focused review. Its distinct contribution is a clinical actionability framework linking biomarker validity to individual discrimination, incremental value, decision impact, implementation, and ultimately patient benefit.

We therefore examine sNfL through the lens of clinical actionability and personalized decision support, considering the evidence required for clinical utility, the conditions necessary for patient-level interpretation, its incremental value beyond established monitoring, its potential role in clinical decisions, and the evidence still needed to show that sNfL-informed management improves patient outcomes. The central question is not simply “Is sNfL useful in MS?”, but rather: “When does sNfL add enough information to change what we should do for the individual patient—and what evidence is required before we act on it?”

## 2 Literature search and review approach

This critical narrative review was informed by structured, purposive searches of PubMed/MEDLINE, with no lower publication-date limit, up to 26 August 2026 and without language restriction. Peer-reviewed publications were considered, including relevant non-English articles when the full text could be obtained and assessed, with translation used when necessary. The principal searches combined “multiple sclerosis” with “neurofilament light chain” or “sNfL” and with three concept blocks: (i) clinical utility, actionability, decision making, management, implementation, monitoring, treatment decisions, or biomarker-guided care; (ii) reference values, percentiles, Z-scores, longitudinal change, variability, standardization, assays, confounders, body mass index, or renal function; and (iii) treatment response, escalation, de-escalation, discontinuation, progression, progression independent of relapse activity (PIRA), glial fibrillary acidic protein, or GFAP.

Targeted supplementary searches identified methodological literature on incremental value, prediction-model performance, decision-curve analysis, and net benefit, as well as named studies, guidance documents, and ongoing implementation trials. Reference lists of key reviews, systematic reviews, consensus statements, and relevant primary studies were examined for additional publications.

Titles and abstracts were assessed by the author, with full-text review when a publication appeared potentially relevant to the review question. Selection was purposive rather than exhaustive. Priority was given to guidance or consensus documents, systematic reviews and meta-analyses, large or longitudinal cohorts, studies assessing individualized interpretation or incremental predictive value, decision-impact studies, and prospective trials or protocols evaluating biomarker-informed care. Small or highly treatment-specific studies were retained only when they addressed a distinct clinical scenario or evidentiary gap. Non-peer-reviewed reports, conference abstracts, preprints, and papers without direct relevance to interpretation, incremental value, decision impact, or outcome benefit were not included.

No review protocol was registered. Because the purpose was critical conceptual synthesis across heterogeneous evidence domains, no duplicate screening, formal risk-of-bias assessment, or quantitative synthesis was undertaken; the methods and findings should not be interpreted as those of a systematic review. The author conducted and reviewed the searches and made all decisions regarding inclusion and interpretation. Generative AI assisted with refinement of search terminology but did not autonomously determine eligibility or evaluate scientific evidence.

Evidence-strength labels used in the figures were assigned qualitatively as part of the narrative synthesis rather than through a formal grading system. The descriptors reflected the overall amount, consistency, and clinical maturity of the available evidence relevant to each stage of the framework, including whether findings had been demonstrated across multiple studies or cohorts and, for the higher levels of actionability, whether evidence addressed incremental information, influence on clinical decisions, implementation in practice, or patient-relevant outcome benefit. These judgments were intended to indicate the relative maturity of the evidence across successive translational stages; no numerical scoring algorithm or formal GRADE assessment was applied.

## 3 From biomarker validity to clinical actionability: what evidence is actually needed?

The clinical value of sNfL cannot be inferred from association alone. Clinical actionability therefore requires evidence across several distinct but interconnected levels.

This distinction is well established in the methodological evaluation of diagnostic and prognostic markers. Evidence that a marker is independently associated with an outcome is not equivalent to evidence that it provides useful individual discrimination; useful discrimination does not establish meaningful incremental value beyond information already available; incremental value does not necessarily translate into improved clinical decision-making; and changing a decision does not establish that the resulting decision improves patient outcomes. Accordingly, evaluation of a new biomarker should consider not only association, but also discrimination, its incremental contribution beyond information already available, and ultimately the consequences of using the biomarker to guide decisions (4, 5, 10–13).

For the purposes of this review, we therefore propose a clinical actionability ladder comprising seven successive levels: analytical validity, biological validity, clinical association, individual discrimination, incremental value, decision impact, and outcome benefit (**Figure 1**). The ladder is not intended as a formal biomarker qualification system. Rather, it is a conceptual framework developed for this review by applying established principles of biomarker evaluation, prediction-model assessment, and decision analysis to the translational development of sNfL in MS. The proposed seven-level structure is author-derived and has not undergone formal validation; it should therefore be regarded as a heuristic framework for organizing and interrogating the evidence rather than as a validated clinical or regulatory instrument. Importantly, progression through the ladder is not automatic: evidence supporting one level does not establish the next. The evidence-strength labels used in the figures are qualitative judgments derived from this narrative synthesis and should not be interpreted as formal GRADE ratings.

**Figure 1:**
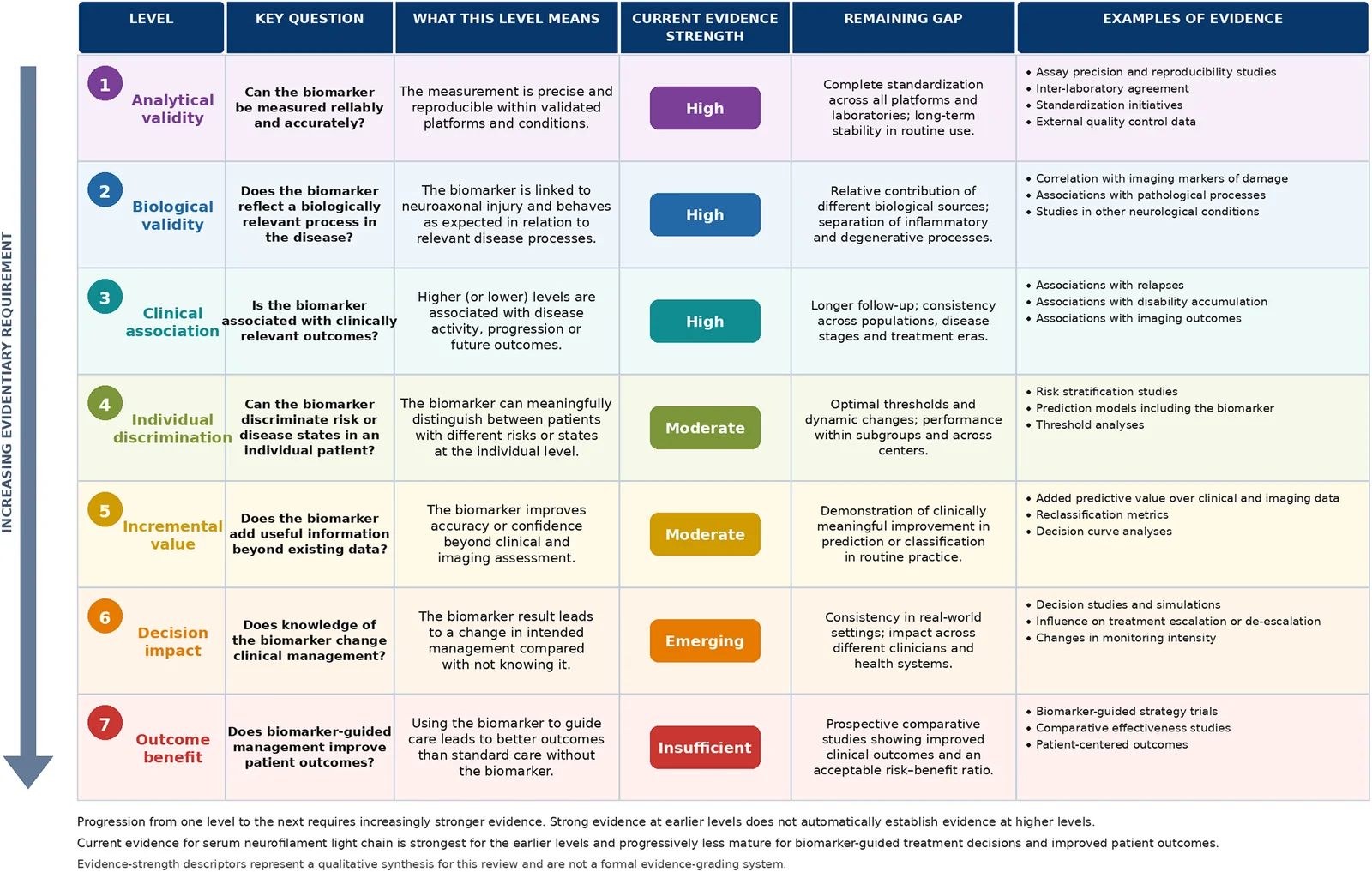
The clinical actionability ladder for serum neurofilament light chain in multiple sclerosis. Proposed conceptual framework illustrating the successive levels of evidence required to translate serum neurofilament light chain (sNfL) from a measurable biomarker into a tool capable of improving clinical care in multiple sclerosis (MS). The ladder distinguishes analytical validity, biological validity, clinical association, individual discrimination, incremental value, decision impact, and outcome benefit. These levels address different questions and should not be considered interchangeable. Association does not establish individual discrimination; individual discrimination does not establish meaningful incremental value; incremental value does not necessarily translate into decision impact; and evidence that biomarker information changes management does not demonstrate that the resulting decision improves patient outcomes. As the consequences of biomarker-informed actions increase, progressively stronger evidence is therefore required. The framework is proposed for this review and is informed by established approaches to biomarker evaluation, prediction-model assessment, and decision analysis (4, 5, 10–13). Evidence-strength labels are qualitative judgments from the narrative synthesis and are not formal GRADE ratings. MS, multiple sclerosis; sNfL, serum neurofilament light chain.

The first three levels establish whether the biomarker signal is credible and clinically relevant. Analytical validity concerns reliable and reproducible measurement; biological validity, whether the measured signal reflects neuroaxonal injury; and clinical association, whether sNfL relates to clinically relevant MS states or outcomes. Evidence at these levels is extensive (2, 3). However, even strong population-level associations do not necessarily imply useful individual discrimination. For clinical decision-making, the relevant question is not only whether patients with disease activity have higher sNfL concentrations on average, but how reliably an individual result distinguishes patients with and without the outcome of interest.

Recent evidence illustrates this gap. In a systematic review and meta-analysis of 28 studies including 11,213 patients, pooled areas under the receiver operating characteristic curve for sNfL discrimination of MS disease activity ranged from 0.61 to 0.71. For clinical relapse, multi-cutoff modeling across the included Simoa-based studies identified an optimal concentration of 10 pg/mL, but discrimination remained modest (sensitivity 0.69, specificity 0.65, AUC 0.72). Substantial heterogeneity in disease-activity definitions, analytical approaches and threshold strategies was present, and half of the studies were considered at high risk of bias, mainly because of *post hoc* threshold selection (14). These findings do not question the biological relevance of sNfL; rather, they illustrate why association and individual classification should not be considered equivalent.

Even useful discrimination would not establish incremental value. In contemporary MS care, sNfL is interpreted alongside relapse history, neurological examination, disability trajectory, MRI findings, treatment exposure and previous disease activity. The relevant question is therefore whether sNfL meaningfully changes the assessment beyond what is already known. A biomarker may remain independently associated with an outcome yet add little to a well-performing reference model. Conversely, modest improvement in global discrimination may still be clinically relevant if the additional information changes risk estimation near a meaningful decision threshold (4, 5, 11). Thus, the incremental value of sNfL is inherently context-dependent: it depends on the information available before the result is known and on the decision that the additional information is intended to inform.

This concern is not merely theoretical. In the study by Moradi et al., clinicodemographic models showed modest predictive performance, but the inclusion of normalized NfL concentrations produced little additional improvement in discrimination for relapse, confirmed disability worsening, or confirmed disability improvement. The findings varied to some extent among treatment groups and outcomes, but the overall incremental contribution of NfL was minimal (15). This illustrates why the prognostic relevance of a biomarker should not be assumed to translate into meaningful improvement over an already informed clinical assessment.

Incremental information, however, is not equivalent to decision impact. A biomarker may improve risk estimation without changing management. Real-world disclosure studies and standardized clinical scenarios show that sNfL information can alter intended management and clinicians’ certainty (6, 16). However, these studies do not establish outcome benefit.

Prospective implementation and strategy trials are now evaluating how sNfL information influences routine decision-making and whether biomarker-informed care improves outcomes (7, 8). Because the available publications are study protocols, they establish that these questions are being tested but do not yet demonstrate decision or outcome benefit.

This distinction between prediction and decision is important because converting a probability into an action requires consideration of the consequences of acting and not acting. Measures such as sensitivity, specificity or AUC do not incorporate those consequences. Decision-analytic approaches, including decision-curve analysis and net benefit, were developed to address this gap between predictive performance and clinical usefulness (10–12). For sNfL, this has a direct practical implication: the evidence required to act on a result should be proportional to the consequences of being wrong. Using an unexpected result to repeat the measurement, investigate alternative causes of neuroaxonal injury or advance MRI surveillance does not require the same degree of certainty as using it to escalate, de-escalate or discontinue long-term disease-modifying therapy.

The highest level of the ladder is therefore outcome benefit. Demonstrating that sNfL changes management is insufficient to establish that sNfL-guided management improves care. This requires prospective comparison of a predefined biomarker-informed strategy with otherwise comparable conventional care, assessing both potential benefits and the consequences of unnecessary investigations or treatment changes, treatment-related harms, patient burden and resource use (11, 13).

Current evidence is therefore asymmetrical across the actionability ladder. It is strongest for analytical and biological validity and for clinical associations with inflammatory activity and prognosis. Evidence for individual discrimination and incremental value is less mature and context-dependent; evidence of decision impact is emerging; and direct evidence that predefined sNfL-informed management strategies improve patient outcomes remains limited. Contrasting expert positions on whether sNfL is ready for routine clinical use further reflect this unresolved translational gap (17, 18). These publications represent expert interpretations of the evidence rather than direct demonstrations of clinical utility and should be understood as such.

At present, sNfL is therefore better regarded as a decision-support biomarker rather than an autonomous treatment-decision biomarker. Its potential value lies in modifying clinical probability and uncertainty within an already informed assessment, with the strength of any resulting action calibrated to the reliability of the signal and the consequences of acting on it.

## 4 Interpretability as a prerequisite for clinical actionability

Clinical actionability presupposes interpretability. Before asking whether a serum neurofilament light chain (sNfL) result adds information or should influence management, a more basic question must be answered: does the measured value represent a biologically meaningful signal in this particular patient under these particular circumstances? This distinction is especially important for sNfL because the measured concentration reflects not only neuroaxonal injury but also patient characteristics, analytical methodology, and timing (1–3, 19).

For decision-support purposes, interpretation can therefore be approached as a sequence of filters rather than through a single threshold (**Figure 2**): analytical comparability, patient-specific biological context, individualized normalization, temporal context, and longitudinal verification. Only after these conditions have been considered should the next question be addressed: whether the result provides clinically relevant information beyond conventional clinical and MRI assessment (1–3, 19).

**Figure 2:**
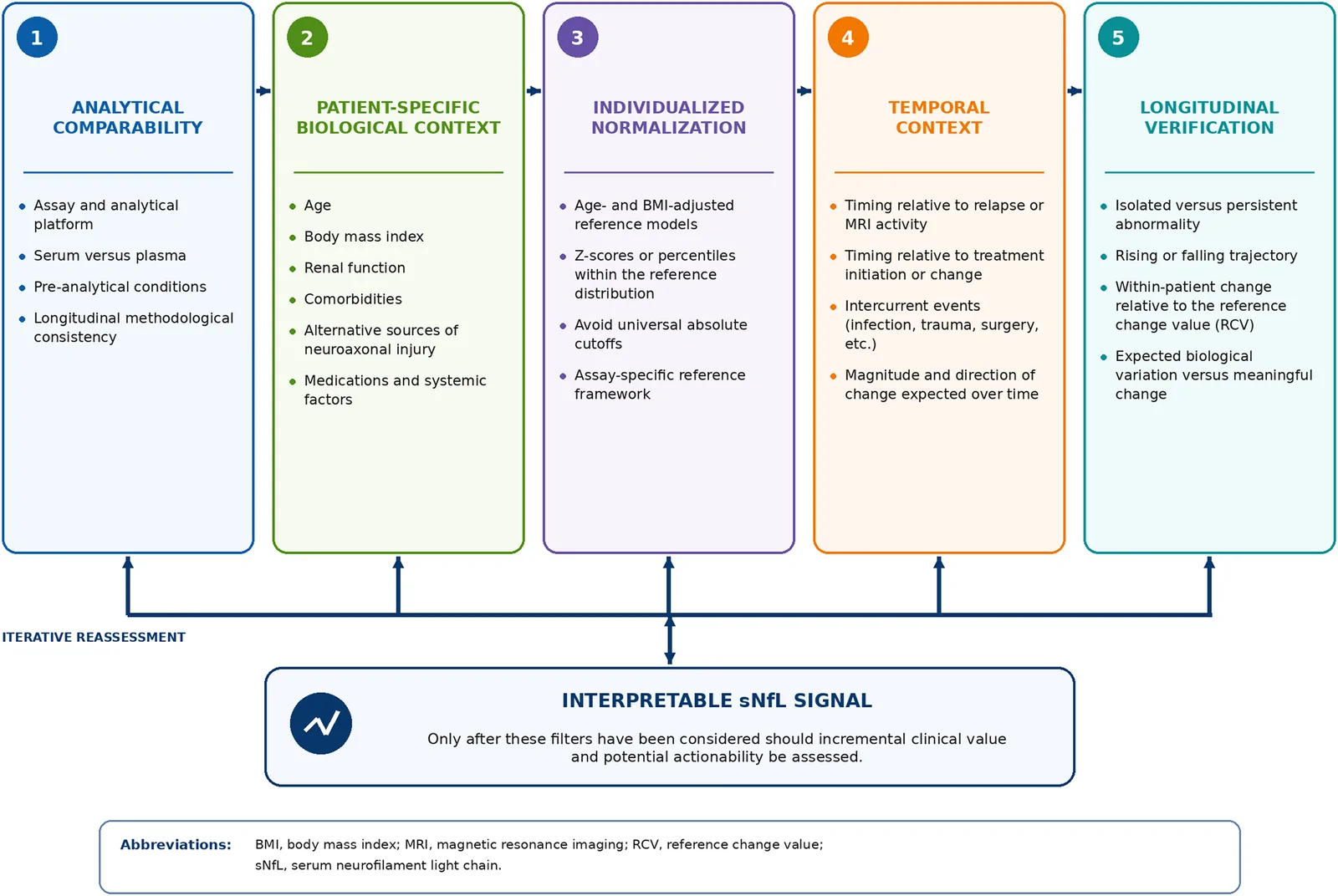
From an sNfL measurement to an interpretable patient-level signal. Proposed sequential framework for determining whether a serum neurofilament light chain (sNfL) result is sufficiently interpretable before its incremental clinical value or potential actionability is considered. Analytical comparability requires attention to assay/platform, sample matrix, pre-analytical conditions and methodological consistency across serial measurements. Patient-specific biological context includes age, body mass index (BMI), renal function, comorbidities and alternative sources of neuroaxonal injury. Individualized normalization places the measured concentration within an appropriate reference distribution, preferably using validated age- and, where available, BMI-adjusted percentiles or Z-scores. Temporal context relates the measurement to recent clinical, MRI, treatment and intercurrent events, whereas longitudinal verification evaluates whether an isolated abnormality represents a persistent or meaningful within-patient change rather than expected variation. These steps do not establish that sNfL should influence treatment; they determine whether the signal is sufficiently credible to proceed to assessment of incremental clinical value. The framework was developed for this review and is informed by evidence on individualized reference values, analytical and biological sources of variation, longitudinal interpretation and clinical implementation of sNfL (1–3, 19). BMI, body mass index; MRI, magnetic resonance imaging; MS, multiple sclerosis; sNfL, serum neurofilament light chain.

### 4.1 Analytical comparability: is the number comparable?

The first filter is analytical: before interpreting a change in sNfL as biological, the measurements must be sufficiently comparable. Increasingly sensitive and automated platforms facilitate routine blood NfL measurement, but absolute concentrations are not necessarily interchangeable across assays and analytical systems; assay-specific validation, appropriate reference intervals, and improved cross-platform standardization therefore remain important prerequisites for clinical implementation (3, 20). Recent real-world data illustrate the distinction between correlation and interchangeability: Elecsys and Simoa measurements obtained from the same samples were strongly correlated, yet showed a marked systematic difference in absolute concentrations, despite broadly similar associations with clinical and MRI variables (21).

For personalized monitoring, the practical implication is straightforward: longitudinal methodological consistency matters more than the nominal value alone. Whenever possible, serial measurements should use the same sample matrix, analytical platform and laboratory procedure, and results should be interpreted using a reference framework validated for that method (2, 3). Otherwise, an apparent within-patient change may partly reflect a change in measurement rather than a change in neuroaxonal injury. This becomes particularly relevant when relatively small longitudinal variations are being considered potentially meaningful.

### 4.2 Biological context: is the signal plausibly attributable to MS?

Once analytical comparability has been established, the next question is whether an abnormal result is biologically plausible for the individual patient and whether MS is the most likely explanation. sNfL is a marker of neuroaxonal injury, not a disease-specific marker. Age is one of its strongest physiological determinants, with concentrations increasing across the lifespan and becoming more variable at older ages. BMI also influences circulating concentrations, with higher BMI generally associated with lower sNfL, at least partly through blood-volume-related effects (22). Renal function is another important modifier: reduced estimated glomerular filtration rate is associated with higher circulating sNfL and becomes particularly relevant in older patients and those with comorbidity (1–3).

The magnitude of some of these effects can be large enough to alter interpretation. In a recent longitudinal real-world RRMS cohort, renal impairment was associated with sNfL elevations of 16.0% in patients with eGFR stage G2 and 67.8% in those with G3–G5, illustrating how an apparently abnormal biomarker signal may be substantially influenced by a non-MS factor (19).

Other sources of neuroaxonal injury—including cerebrovascular disease, traumatic brain injury, seizures, infection and other neurological or systemic conditions—may likewise increase circulating NfL independently of MS activity (3). These factors need not be converted into an exhaustive exclusion checklist; rather, they change the probability that a given sNfL abnormality is attributable to MS. The clinically relevant question is therefore not simply “Is sNfL elevated?”, but “Is this elevation unexpected for this patient, and is MS the most plausible explanation?”

### 4.3 From absolute concentrations to individualized normalization

Between-person variability makes universal absolute cut-offs intrinsically problematic. Reference models provide a more individualized interpretation by asking how unusual a measured concentration is for a person with relevant characteristics similar to those of the patient.

Large normative datasets have enabled age-adjusted percentiles and Z-scores, with some models additionally incorporating BMI. Benkert et al. derived reference percentiles from more than 10,000 samples from 5,390 healthy individuals and showed that age- and BMI-adjusted sNfL Z-scores were associated with subsequent MS disease activity, including among patients without contemporaneous clinical or MRI evidence of activity (1). The importance of physiological normalization is further illustrated across the age spectrum: in pediatric populations, the relationship between age and sNfL is markedly non-linear, and age-adjusted Z-scores showed larger effect sizes than absolute concentrations when comparing healthy individuals with patients with neurological disorders (23).

More recent longitudinal data provide direct evidence that adjusted measures may outperform absolute cut-offs. In a cohort of 447 patients with relapsing MS receiving fingolimod, age- and BMI-adjusted Z-scores or percentiles more accurately identified associations with clinical and MRI disease activity than absolute concentration thresholds. Approximately 20% of patients were classified discordantly by the two approaches, illustrating the potential for both false-positive and false-negative interpretation when universal absolute cut-offs are used (24). The rationale for normalization is simple but important: the clinically relevant question changes from “Is 12 pg/mL high?” to “Is this value unexpectedly high for this individual?”

Importantly, improved normalization should not be conflated with improved standalone discrimination. Absolute thresholds, percentile-based approaches, and adjusted Z-scores have all shown limited discrimination of MS disease activity (14); normalization should therefore be regarded as improving interpretability rather than establishing sufficient accuracy for standalone classification or treatment decisions.

Normalization should therefore be understood as a means of reducing interpretative noise, not as a way of converting a continuous biomarker into a definitive binary state. Importantly, population-based normalization and longitudinal individualization address different sources of uncertainty: the former contextualizes a value relative to an appropriate reference population, whereas the latter asks whether change within the same patient exceeds expected analytical and biological variation. These approaches should therefore be regarded as complementary rather than interchangeable (19).

### 4.4 Temporal context: when was the sample obtained?

An sNfL measurement also carries a time dimension. Blood NfL does not provide an instantaneous snapshot of ongoing disease activity but reflects recent and ongoing neuroaxonal injury over a biological window extending across weeks and possibly months (25, 26). Concentrations may rise in relation to inflammatory activity and remain elevated beyond the event that generated the signal. Sampling in proximity to a relapse, new MRI lesion, trauma, infection, or another acute neurological event can therefore affect interpretation of both the absolute value and its subsequent trajectory (2, 3).

Timing is particularly important when assessing treatment response. An elevated value shortly after treatment initiation may partly reflect injury occurring before treatment or before full therapeutic effect, whereas persistent or newly rising values later in follow-up raise a different question. Thus, the same numerical result may have different meanings at baseline, shortly after inflammatory activity, during treatment stabilization, and after prolonged clinical and radiological stability (25, 27).

### 4.5 From isolated values to within-patient trajectories

Individualization ultimately requires moving beyond whether a single result lies inside or outside a reference range. Once serial measurements are available, the patient’s own previous trajectory becomes an increasingly important reference. High-frequency longitudinal assessment has shown that individual patients may establish relatively stable low sNfL levels during disease control, whereas transient or sustained increases can accompany clinical or MRI activity (28). A persistently high but stable value, a substantial decline after treatment, and a progressively rising value may therefore carry very different information, even when interpreted against the same population reference range.

Recent evidence illustrates the potential value of formalizing this within-patient perspective. In a longitudinal real-world RRMS cohort, substantial between-patient heterogeneity was observed, with an intraclass correlation coefficient of 0.536, indicating that a considerable proportion of sNfL variability reflected stable differences between individuals. In the same cohort, a reference change value (RCV) approach designed to identify changes exceeding expected analytical and biological variation was more strongly associated with inflammatory activity than a population-based threshold. RCV elevation was associated with active MRI (OR 4.93, 95% CI 2.49–9.77) and clinical relapse (OR 6.48, 95% CI 2.75–15.25), whereas elevation according to the population-based threshold was not significantly associated with either outcome (19). Although negative predictive values exceeded 91% in that cohort, such values are prevalence-dependent and do not establish a general rule-out threshold across clinical settings. The findings require external validation and do not establish RCV as a treatment threshold, but they illustrate an important principle: for longitudinal monitoring, meaningful deviation from an individual’s previous trajectory may sometimes carry more information than crossing a population-derived boundary.

A similar principle applies to normalized trajectories, which may provide information beyond a single normalized measurement (1, 27). However, the optimal longitudinal metric—absolute or percentage change, Z-score trajectory, reference change value, or another within-patient measure—remains unresolved, and available thresholds require further validation (19, 27).

The purpose of these interpretative steps is not to create a more sophisticated definition of “high sNfL”, but to determine whether an observed signal is sufficiently credible to enter the clinical decision process. Only then does the question change from *“Can this result be interpreted?”* to *“Does it tell us something clinically important that we did not already know?”* That is the question addressed in the next section.

## 5 Where does sNfL really add information?

Once an sNfL result has been shown to be interpretable in the individual patient, the next question is whether knowing the result meaningfully changes what is already known from clinical assessment and MRI. The relevant question is therefore not whether sNfL can classify disease activity by itself, but what it adds to the information already available.

### 5.1 Incremental value is context-dependent

Several longitudinal studies suggest that sNfL can refine risk estimates beyond conventional clinical and MRI assessment. Importantly, however, statistical independence in a multivariable model should not automatically be equated with clinically meaningful incremental value. The relevant question is whether access to the biomarker changes the estimated probability of a clinically relevant outcome sufficiently to meaningfully alter risk assessment or decision-making.

Age- and BMI-adjusted Z-scores provide important evidence in this regard. In the Swiss Multiple Sclerosis Cohort, elevated sNfL Z-scores predicted subsequent clinical or MRI activity even among patients fulfilling contemporaneous NEDA-3 criteria; a Z-score >1.5 was associated with greater risk of disease activity during the following year (OR 2.66, 95% CI 1.08–6.55), and the overall findings were reproduced in an independent Swedish registry cohort (1). Thus, patients who appear similar according to conventional monitoring may nevertheless carry different levels of biological risk.

Real-world observations extend this concept. Among treated patients who had fulfilled NEDA-3 during the preceding year, Malucchi et al. found that an elevated sNfL measurement was strongly associated with loss of NEDA-3 during the subsequent 12 months (OR 10.74, 95% CI 4.34–26.57), whereas the association was no longer detected during later follow-up intervals (29). This temporal pattern suggests that the additional information provided by sNfL may be particularly relevant to proximal biological risk, rather than functioning as a durable prognostic label.

Studies explicitly evaluating model performance provide a more stringent assessment of incremental value. In the CLIMB cohort, Bose et al. evaluated 144 patients early after MS onset and examined 10-year clinical and MRI outcomes. Adding baseline sNfL and sGFAP to a clinical model increased the area under the receiver operating characteristic curve for subsequent SPMS from 0.73 to 0.77 (P = 0.021), while adding 1-year biomarker measurements to the model already containing baseline biomarkers increased it further to 0.79, although this additional improvement was not statistically significant (P = 0.15). Biomarkers also improved explained variance for subsequent EDSS and MRI outcomes, with partly different contributions across outcomes: sGFAP was more closely associated with later clinical progression, whereas sNfL contributed more strongly to MRI measures of lesion burden and brain tissue loss (30). These findings illustrate that statistically measurable incremental value may nevertheless be modest in clinical terms.

The magnitude of the additional information should not be overstated. Brummer et al. studied patients without clinical relapses, new T2 lesions or gadolinium-enhancing lesions and found that, among those with increasing whole-brain lesion volume, sNfL remained independently associated with subsequent EDSS progression (OR 1.344, 95% CI 1.038–1.739). Nevertheless, its individual discriminatory performance was modest (AUC 0.669; sensitivity 44.4%; specificity 85.9%) (31). This apparent tension is informative rather than contradictory: a biomarker may carry independent prognostic information without being sufficiently discriminative to determine an individual treatment decision.

Taken together, these studies support a narrower but clinically relevant conclusion: sNfL can reduce residual uncertainty after conventional assessment, but the magnitude and clinical relevance of that reduction depend on the context, the outcome being predicted, and the information already available.

### 5.2 Discordance as the potentially informative setting

The clinical relevance of incremental information depends not only on the prognostic information carried by sNfL, but also on what is already known from clinical and MRI assessment. Its contribution is therefore unlikely to be distributed equally across all combinations of clinical, MRI and biomarker findings. When an unequivocal relapse or new MRI activity is accompanied by elevated sNfL, the biomarker provides biologically coherent support for recent or ongoing neuroaxonal injury in the context of an already recognized active state but may contribute relatively little new information. Similarly, concordantly reassuring clinical, MRI, and sNfL findings may strengthen confidence in apparent stability without necessarily changing management (2, 25).

From an incremental-information perspective, discordant findings may therefore be particularly informative. As illustrated in **Figure 3**, the incremental contribution of sNfL can be conceptualized according to its concordance or discordance with the clinical and MRI assessment. An appropriately interpreted elevated or rising sNfL result in a patient who appears clinically and radiologically stable challenges the existing assessment and may identify residual risk not captured by intermittent conventional monitoring. Conversely, a non-elevated sNfL result in the presence of convincing clinical or MRI activity generates a different form of discordance and should prompt consideration of the biological domain that sNfL does—and does not—capture (1, 2, 14, 19, 29).

**Figure 3:**
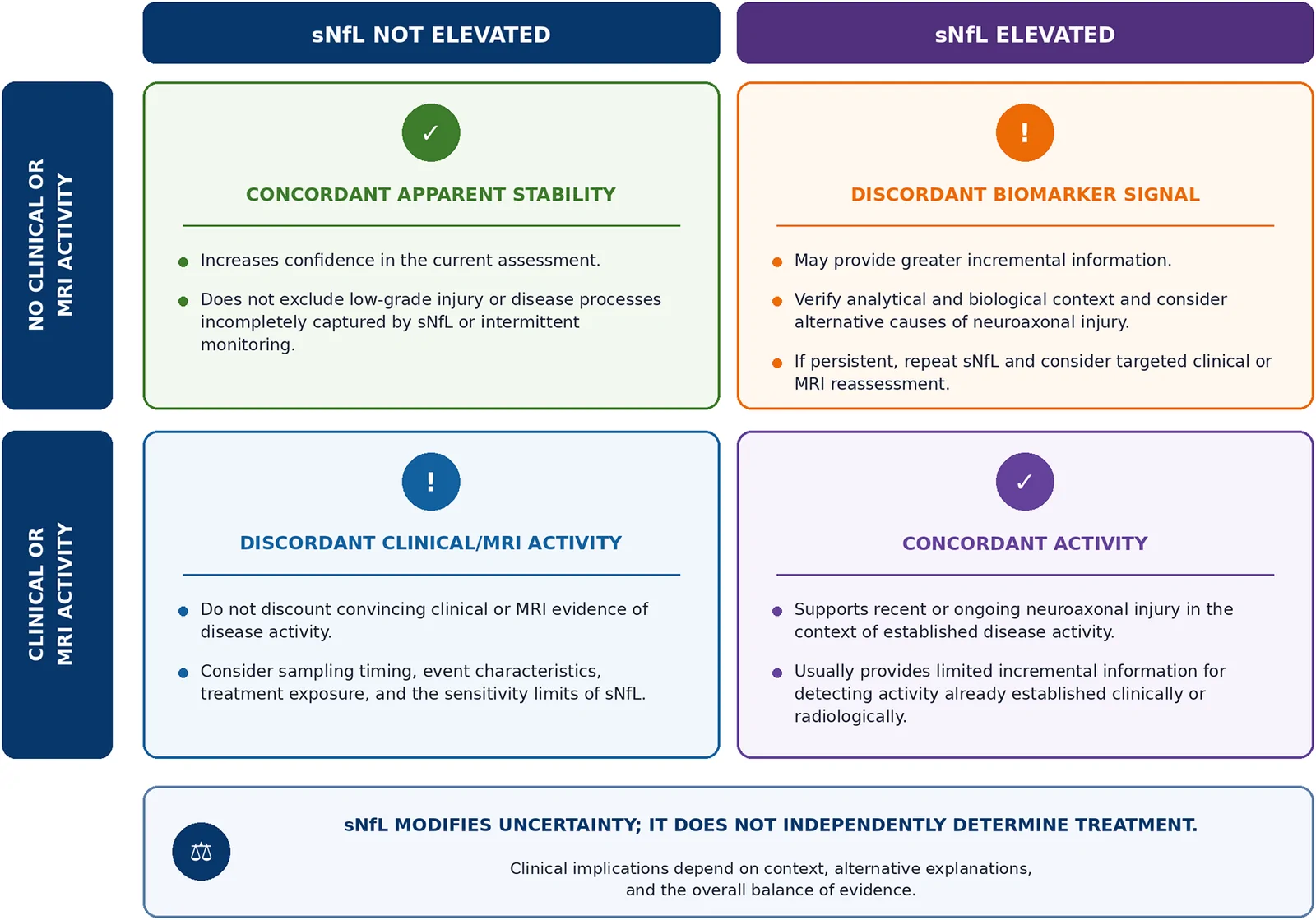
Discordance as a source of incremental information from serum neurofilament light chain in multiple sclerosis. Conceptual framework illustrating how an appropriately interpreted serum neurofilament light chain (sNfL) result may modify information already provided by clinical and magnetic resonance imaging (MRI) assessment. Concordant findings mainly reinforce an existing interpretation, whereas discordance may provide greater incremental information. Elevated sNfL despite apparent clinical and MRI stability may increase suspicion of otherwise unrecognized neuroaxonal injury but should initially prompt verification, consideration of alternative causes and targeted reassessment rather than automatic attribution to uncontrolled MS. Conversely, non-elevated sNfL should not override convincing clinical or MRI evidence of disease activity. Concordantly reassuring findings may increase confidence in apparent stability but do not establish absence of clinically relevant disease evolution or of disease processes incompletely captured by sNfL. The framework therefore conceptualizes sNfL as a context-dependent modifier of clinical probability and uncertainty rather than an autonomous treatment trigger. The framework synthesizes evidence on individualized interpretation, discriminatory performance, discordance, and longitudinal risk (1, 2, 14, 19, 29). MRI, magnetic resonance imaging; MS, multiple sclerosis; sNfL, serum neurofilament light chain.

Importantly, discordance should be regarded as a signal requiring explanation rather than as evidence that one source of information is necessarily more valid than another. Its value lies in challenging the existing assessment and redirecting attention, not in providing an autonomous answer; the proportionate clinical responses to each discordant pattern are considered in Section 6 (2, 19).

### 5.3 Apparent stability with elevated or rising sNfL

The clinically and radiologically stable patient may be one of the settings in which sNfL provides its greatest incremental information. Evidence from NEDA-3 populations indicates that elevated sNfL can identify patients at greater short-term risk of subsequent activity despite an initially reassuring conventional assessment (1, 29).

The importance of these observations lies less in another demonstration of prognostic association than in the discordance they reveal. An elevated or rising sNfL does not prove occult MS activity; rather, it challenges the completeness of an otherwise reassuring assessment.

Thus, in the apparently stable patient, sNfL may be most useful not as an alternative definition of NEDA or disease activity, but as a means of determining how confident the clinician should be in apparent stability.

### 5.4 Conventional evidence of activity with non-elevated sNfL

The opposite discordance requires an asymmetric interpretation. A normal or decreasing sNfL value cannot negate convincing clinical or MRI evidence of MS activity. The extent to which neuroaxonal injury is reflected in sNfL depends on its magnitude, location and timing, as well as on treatment exposure and individual biological factors; therefore, not every clinically meaningful inflammatory event will necessarily result in an elevated sNfL concentration (2, 3).

When the pre-test probability of disease activity is already high because of an unequivocal relapse or new MRI activity, a non-elevated sNfL result provides limited incremental reassurance. This limitation is even more important when the concern is progression rather than focal inflammatory activity; the implications for PIRA, GFAP and multimodal monitoring are considered in Section 7.

### 5.5 Confirmation is useful, but it is not the same as incremental value

Concordant results may reinforce an existing interpretation, but the relevant counterfactual question is whether the clinical assessment, surveillance strategy or management decision would have been different without the sNfL result. If the answer is no, the biomarker may still provide biologically meaningful support, but its incremental contribution to that particular decision is limited (4, 5, 11).

The same sNfL result can therefore add very different amounts of information in different patients. Its value depends on prior clinical and MRI evidence, the previous biomarker trajectory and the question being asked. sNfL is consequently better understood as a context-dependent modifier of clinical probability and uncertainty than as a binary classifier of disease activity.

The next question is what proportionate clinical response—if any—should follow when the biomarker meaningfully changes the assessment of an individual patient.

## 6 From information to action: clinical scenarios

Once an sNfL result is interpretable and provides information beyond conventional assessment, the next question is whether that information should modify management. Actionability does not mean that every abnormal result should trigger a treatment change. The appropriate response depends on the clinical context, the direction and persistence of the biomarker signal, its concordance with other evidence and, critically, the consequences of acting incorrectly (2).

A practical approach is therefore not to apply a universal threshold-to-treatment algorithm, but to consider clinical scenarios in which sNfL modifies uncertainty and informs the next proportionate step. Frequently, that step will be verification or targeted reassessment rather than treatment modification. **Table 1** summarizes this context-dependent approach.

**Table 1:** Context-dependent interpretation and potential clinical responses to serum neurofilament light chain in multiple sclerosis.

Clinical scenario	What sNfL may add	What sNfL should not be used to conclude	Suggested clinical response	Key evidence
Baseline assessment before DMT initiation or modification	Provides an individualized reference for subsequent longitudinal interpretation, particularly when expressed using age- and BMI-adjusted Z-scores or percentiles. Elevated baseline sNfL may also identify greater risk of subsequent disease activity or structural brain changes.	An isolated baseline value does not establish the need for a particular DMT or determine treatment intensity.	Interpret the result using an appropriate reference model and retain it as the starting point for evaluating the subsequent biological trajectory. Base treatment selection on the integrated clinical, MRI and patient context.	1–2, 32.
Early assessment after DMT initiation or modification	A decreasing longitudinal trajectory may provide evidence of biological treatment response. Failure of sNfL or its Z-score to decrease may identify residual risk not fully captured by conventional first-year response measures.	An unfavorable trajectory does not, by itself, establish treatment failure or justify treatment escalation. Conversely, reaching the normal range does not establish complete disease control.	Review the magnitude and timing of change, clinical and MRI activity, sampling context, adherence where relevant and alternative causes of neuroaxonal injury. Repeat measurement when appropriate and require corroborating evidence before treatment escalation.	27, 33–34.
Apparently stable disease with elevated or rising sNfL	An elevated sNfL or an unfavorable longitudinal trajectory may identify residual biological risk not captured by intermittent conventional monitoring.	A single elevated value does not establish uncontrolled MS, occult MS activity or treatment failure.	Verify the result and its analytical, biological and temporal context; consider non-MS causes of neuroaxonal injury; review the longitudinal trajectory and consider repeat measurement, closer surveillance or targeted clinical or MRI reassessment.	1–2, 27, 29, 35.
Clinical or MRI activity with elevated sNfL	Provides biologically coherent support for recent or ongoing neuroaxonal injury accompanying recognized disease activity.	Concordance does not necessarily provide substantial incremental information or independently determine treatment escalation.	Base management on the integrated clinical and MRI assessment; use sNfL as supportive rather than determinative evidence.	1–2, 36.
Clinical or MRI activity with non-elevated sNfL	Provides limited reassurance because the timing, magnitude or location of neuroaxonal injury and individual biological factors may influence the circulating biomarker response.	A non-elevated sNfL should not be used to dismiss or downgrade convincing clinical or MRI evidence of disease activity.	Prioritize disease-specific clinical and MRI findings and consider the timing of sampling and longitudinal biomarker behavior.	2, 14.
Suspected progression or PIRA without overt inflammatory activity	May identify increased neuroaxonal injury preceding some relapse-independent disability-worsening events, although associations are heterogeneous and sGFAP may capture complementary progression-related biology.	Normal or stable sNfL does not exclude relapse-independent disability accumulation or establish the absence of progression.	Maintain longitudinal clinical and MRI assessment; interpret sNfL cautiously and consider complementary biological information, particularly sGFAP, where appropriate.	37–41.
DMT de-escalation or discontinuation	Serial sNfL after treatment withdrawal may provide additional information on subsequent risk; higher or increasing post-discontinuation values have been associated with disability worsening and new MRI activity.	Low or stable sNfL before withdrawal does not establish that discontinuation is safe. The absence of a differential biomarker signal does not establish the absence of clinically relevant disease evolution or PIRA.	Base discontinuation decisions on the overall clinical context rather than sNfL alone. If treatment is withdrawn, consider longitudinal sNfL as an adjunct to clinical and MRI surveillance, not as an independent rule for restarting treatment.	42–43.

Proposed responses represent decision-support strategies based on current evidence and clinical reasoning rather than validated biomarker-driven treatment algorithms. The strength of evidence required to act on sNfL should increase with the potential consequences of the intervention.

BMI, body mass index; DMT, disease-modifying therapy; MRI, magnetic resonance imaging; MS, multiple sclerosis; PIRA, progression independent of relapse activity; sGFAP, serum glial fibrillary acidic protein; sNfL, serum neurofilament light chain.

For example, in a clinically stable patient with an unexpected sNfL increase and no recent MRI evidence of activity, the biomarker would not by itself justify treatment escalation, but a persistent and otherwise unexplained increase could reasonably prompt repeat testing, review of potential confounders, and earlier or targeted MRI. Conversely, when clinical or MRI activity is already present, elevated sNfL may strengthen the inference of ongoing neuroaxonal injury and support reassessment of treatment adequacy (1, 2), whereas a low sNfL result should not override unequivocal clinical or radiological activity. In each case, sNfL modifies the level of concern and the intensity of reassessment rather than functioning as an autonomous treatment trigger.

### 6.1 Baseline assessment: establishing a reference, not a treatment threshold

A baseline sNfL measurement obtained before DMT initiation or modification can provide a useful reference for subsequent longitudinal interpretation. Individualized reference models, including age- and BMI-adjusted Z-scores or percentiles, can improve interpretation beyond unadjusted absolute concentrations (1). Baseline sNfL has also been associated with subsequent structural brain changes, including accelerated brain volume loss (32).

Its main practical purpose, however, is not to assign a patient to a treatment category. Current evidence does not support selecting a specific DMT solely because an isolated baseline sNfL value exceeds a particular threshold. Rather, an appropriately interpreted baseline measurement provides a starting point against which the patient’s subsequent biological trajectory can be judged.

### 6.2 Early treatment response: is the trajectory moving in the expected direction?

Reductions in sNfL after DMT initiation have been observed across therapeutic settings and may provide a longitudinal marker of biological treatment response. In a prospective observational cohort of 85 treatment-naïve patients who completed 18 months of follow-up, sNfL decreased after the initiation of high- and moderate-efficacy DMTs, and its reduction correlated with improvements in EDSS and the 9-Hole Peg Test. However, treatment allocation was not randomized, sNfL was measured using an ELISA-based assay and attrition was substantial, limiting causal and broader clinical interpretation (33).

Changes in individualized sNfL values may also complement established treatment-response measures. Failure of the sNfL Z-score to decrease during the first treatment year was associated with subsequent disease activity, including among patients achieving NEDA-3 during the first year and within otherwise favorable treatment-response score strata (27).

Evidence from randomized trials further supports the responsiveness of NfL to treatment at the group level. In a meta-regression of 15 randomized controlled trials including 6,814 patients, treatment effects on NfL were moderately associated with treatment effects on annualized relapse rate, new or enlarging T2 lesions and confirmed disability worsening, but not with percentage brain volume change (34). These trial-level associations support NfL as a marker of biological treatment response, but should not be extrapolated to establish individual-level treatment failure or to justify treatment escalation.

However, an unfavorable sNfL trajectory is not synonymous with treatment failure. An unexpected trajectory should prompt reassessment of clinical and MRI activity, sampling context, adherence where relevant, and alternative causes of neuroaxonal injury, with repeat measurement when appropriate. Trajectory and context are more informative than whether a single follow-up value has crossed into the “normal” range, and treatment escalation based solely on an unfavorable sNfL trajectory would exceed current evidence.

### 6.3 Apparent stability with elevated or rising sNfL: look harder before treating harder

An unexpectedly elevated sNfL or an unfavorable longitudinal trajectory in a patient who appears clinically and radiologically stable may identify residual risk not captured by conventional monitoring (1, 27, 29).

The first response should be to verify and explain the signal, not automatically escalate treatment. Once the analytical, biological and temporal factors described in Section 4 have been considered, persistent unexplained elevation may reasonably prompt repeat sNfL measurement, closer clinical surveillance or earlier MRI reassessment (2). Marked elevation may also reflect neurological injury unrelated to MS. A recent case report described increasing sNfL in a patient initially considered to have PIRA in whom the clinical deterioration was subsequently attributed to acquired hepatocerebral degeneration, illustrating the need to investigate alternative explanations for an unexpected biomarker signal (35).

An abnormal sNfL result may justify looking harder before it justifies treating harder.

### 6.4 Clinical or MRI activity with non-elevated sNfL: do not allow the biomarker to veto stronger evidence

The converse scenario requires an asymmetric response. A non-elevated sNfL cannot reliably exclude inflammatory MS activity and should not override an unequivocal relapse or convincing new MRI activity. This follows from the imperfect sensitivity and discriminatory performance of sNfL and from the influence of the timing, magnitude and location of neuroaxonal injury on its circulating concentration (2, 14).

Management should therefore remain driven by the totality of clinical and imaging evidence.

### 6.5 Suspected progression without overt inflammatory activity: recognize what sNfL cannot exclude

Associations between sNfL and relapse-independent disability worsening are heterogeneous, and a normal or stable sNfL value cannot exclude gradual disability accumulation or PIRA (37, 41). Suspected progression should therefore continue to be evaluated through longitudinal clinical and MRI assessment rather than being discounted because the biomarker is reassuring.

The biological basis of this limitation and the potential complementary contribution of GFAP are considered in Section 7. For present purposes, the practical message is sufficient: sNfL should not be used as a biochemical substitute for detecting progression.

### 6.6 De-escalation and treatment discontinuation: absence of a signal is not evidence of safety

The risk of false reassurance becomes particularly important when considering DMT de-escalation or discontinuation. Current evidence does not establish that low or stable sNfL before treatment withdrawal identifies patients in whom discontinuation is safe.

In a recent retrospective study of previously stable patients receiving moderate-efficacy DMTs, discontinuation was associated with increased risks of confirmed disability progression and PIRA at two years despite no significant between-group differences in relapses, MRI activity, NEDA-3 or sNfL levels (43). Importantly, this study was not designed to establish the predictive performance of sNfL for treatment withdrawal; rather, it illustrates that the absence of a differential biomarker signal does not establish the absence of clinically relevant disease evolution after discontinuation.

Longitudinal changes after withdrawal may nevertheless provide additional information. In patients discontinuing DMT after a period of clinical stability, higher or increasing sNfL after treatment cessation was associated with subsequent disability worsening and new MRI activity, whereas pre-discontinuation sNfL was not associated with subsequent disease activity (42). However, the thresholds evaluated were empirically derived, and whether biomarker-guided reinitiation of treatment improves outcomes remains unproven.

sNfL may therefore contribute to post-withdrawal surveillance, but neither a reassuring baseline value nor a subsequent biomarker change should currently function as an independent treatment rule. Decisions to de-escalate, discontinue or restart therapy must remain integrated with the broader clinical and radiological context.

### 6.7 Matching the action to the strength of the evidence

Across these scenarios, one principle is consistent: the evidentiary threshold for acting on sNfL should increase with the potential consequences of the action, as operationalized across the clinical scenarios summarized in **Table 1**. Repeat testing, earlier MRI, or closer surveillance may reasonably follow a weaker signal; treatment escalation, de-escalation, or discontinuation requires substantially stronger corroborating evidence (10–13).

The same proportionality principle should also account for the consequences of not acting. In some settings, disregarding a persistent and otherwise unexplained sNfL signal could delay the detection of subclinical disease activity or postpone appropriate treatment reassessment. The relevant comparison is therefore not between action and no risk, but between the potential harms of unnecessary intervention and those of delayed recognition or response. This reinforces the role of sNfL as a trigger for proportionate verification and reassessment when the evidence is insufficient to justify treatment modification itself.

This distinction helps define the current clinical position of sNfL. The evidence supports it more strongly as a decision-support biomarker, modifying the information on which a decision is based, than as a treatment-decision biomarker capable of independently determining that decision (2, 6, 16).

This leads to a deliberately asymmetric rule for present-day practice: abnormal sNfL may justify looking harder, whereas normal sNfL should not, by itself, justify looking less (2, 14, 37, 41).

## 7 Why clinical actionability may require complementary information

### 7.1 PIRA exposes the limits of a single biomarker

The limits of sNfL are not exclusively analytical or interpretative; they are also biological. Disability accumulation in MS can occur through mechanisms that are only partly reflected by a biomarker of neuroaxonal injury, particularly when overt inflammatory activity is absent. This limitation cannot necessarily be overcome by refining thresholds, normalization or longitudinal interpretation.

Recent evidence illustrates the partly distinct information provided by markers of neuroaxonal and astroglial injury. In a longitudinal cohort using individualized reference models, elevated sGFAP was associated with subsequent PIRA, whereas elevated sNfL was not. Conversely, elevated sNfL was associated with subsequent relapses, whereas sGFAP was not (40). These findings should not be interpreted as establishing a simple biological division between an “inflammatory” sNfL signal and a “progression” GFAP signal. Rather, they suggest that the two biomarkers may capture partly different and overlapping dimensions of MS biology.

Complementary evidence comes from a prospective multicenter cohort of patients with progressive MS in which all confirmed disability progression events occurred independently of relapses. Particularly high GFAP levels were associated with greater progression risk in PPMS, and the combination of high GFAP with low NfL identified patients at particularly high risk. This finding illustrates that a relatively low NfL signal does not reliably exclude clinically relevant relapse-independent progression (39).

At the same time, the relationship between sNfL and relapse-independent disability worsening is not absent, but context-dependent. In two large prospective cohorts, NfL elevation preceded confirmed disability worsening occurring without clinical relapses by approximately 1–2 years (37). Importantly, the authors distinguished this outcome from strictly defined PIRA because the absence of clinical relapses did not imply the absence of MRI or other inflammatory disease activity. In the subset with available MRI data, clinical or MRI activity was present in a substantial proportion of evaluable visits preceding disability worsening, and adjustment for this activity attenuated some of the associations with NfL. An association between sNfL and disability worsening occurring without relapses should therefore not automatically be interpreted as evidence that sNfL captures progression independently of inflammatory activity.

This interpretation is consistent with a recent systematic review of blood NfL and GFAP in relation to PIRA and PIRA-like outcomes. Associations with sNfL were variable and context-dependent, whereas those with GFAP were more consistent, particularly when overt inflammatory activity was limited or suppressed. Substantial heterogeneity in outcome definitions, biomarker measurements and treatment context precluded quantitative synthesis (41).

The practical implication is that the limitations of sNfL in detecting relapse-independent progression may not be resolved simply by improving the interpretation of sNfL itself. Some clinically relevant disease biology may remain incompletely captured by sNfL and require complementary biological and non-biological information.

### 7.2 From a single biomarker to complementary biological information

The limitations of sNfL argue against expecting further refinement of a single marker to provide a complete representation of clinically relevant MS biology. A more plausible direction is to combine signals that provide partly non-redundant information. This should not mean replacing one supposedly universal biomarker with another: current evidence supports complementarity rather than substitution (41).

The differential associations of sGFAP and sNfL with PIRA and relapses illustrate this principle (40). Complementary evidence from a multicenter cohort of patients assessed close to disease onset showed that combined sNfL and sGFAP profiles identified groups with different risks of relapse-associated worsening, PIRA and disability accumulation, although the observational design does not establish that biomarker-based treatment selection improves outcomes (44). Evidence from progressive MS also suggests that biomarker associations may vary across disease phenotypes. In a large progressive MS cohort, the NfL Z-score was associated with subsequent confirmed disability worsening in PPMS but not in the overall progressive MS population, whereas GFAP showed a partly different pattern of associations (45). In other settings, sGFAP has contributed more strongly to the prediction of clinical progression, whereas sNfL has been more closely associated with MRI measures of lesion burden and brain tissue loss (30, 38).

The same principle applies more broadly to multimodal monitoring. Clinical examination, MRI and fluid biomarkers interrogate different aspects of disease and should not be expected to provide interchangeable information. The objective of integration is not simply to accumulate more measurements, but to determine whether complementary information reduces clinically relevant uncertainty beyond what was already known.

The goal should therefore not be to identify a single biomarker capable of declaring whether MS is “controlled”. A more realistic objective is to determine which biological question each marker can answer, in which clinical context, and what uncertainty remains after the result is known.

A richer biological profile is not automatically a more actionable one; the clinical value of multimarker and multimodal strategies will depend on whether the complementary information meaningfully reduces uncertainty beyond established monitoring.

## 8 Discussion and future evidence requirements

The evidence reviewed above supports the biological and prognostic validity of sNfL and its potential to provide incremental information beyond conventional clinical and MRI monitoring. However, the final step towards clinical actionability remains insufficiently demonstrated: whether incorporating sNfL into a predefined management strategy improves patient-relevant outcomes compared with otherwise similar care without biomarker-informed management.

Three propositions emerge from this synthesis. First, patient-level interpretability is necessary but not sufficient for clinical actionability. Second, incremental value is a counterfactual property: it depends on what was known before the biomarker result and whether that result changes clinically relevant uncertainty. Third, the evidentiary threshold for acting on sNfL should rise with the potential consequences of the action. These propositions distinguish an actionability framework from a general implementation guide and provide testable requirements for future biomarker-informed management strategies.

Evidence is beginning to move beyond association towards decision impact. In a prospective implementation study including 166 clinical assessments, disclosure of sNfL results changed the intended clinical decision in 19.3% of cases. The impact was greater when sNfL had been requested because of new symptoms, with decisions changing in 38% of such assessments. Higher sNfL concentrations were associated with a greater likelihood of decision change, and clinicians’ certainty increased after disclosure of the result (6). These findings demonstrate that sNfL can influence clinical reasoning, but a biomarker-induced investigation, change in surveillance or treatment modification may be appropriate, unnecessary or potentially harmful.

The ongoing NeuroFilMS study addresses implementation from a real-world perspective. This prospective, multicenter study embedded in the German MS Registry plans to enroll 1,500 patients with relapsing MS, with randomized allocation in a 2:1 ratio to immediate or delayed reporting of sNfL results to treating physicians. This design will allow prospective evaluation of whether the availability of sNfL influences diagnostic and therapeutic decision-making in routine care, including treatment initiation, escalation and modification (8). Importantly, NeuroFilMS is a study protocol, and no effect on decisions or outcomes has yet been demonstrated. Its primary endpoint concerns the association between longitudinal sNfL and clinical and MRI disease activity, whereas the clinical use of sNfL in decision-making is a secondary endpoint. The study should therefore provide important evidence on longitudinal biomarker behavior, implementation and decision impact, but it is not a definitive test of whether sNfL-informed management improves patient outcomes.

A further translational step is to define how different biomarker patterns could be linked to clinical responses. A modified Delphi study proposed treatment-decision algorithms incorporating normalized sNfL values into scenarios involving treatment initiation, escalation, horizontal switching and de-escalation. The algorithms were designed to support integration of sNfL with clinical and MRI information and explicitly allowed physicians and patients to override the proposed recommendations (46). They help make the decision process explicit and provide a standardized intervention that can be tested prospectively. However, they are expert-derived and non-binding, and their clinical benefit has not been established.

An alternative exploratory framework has combined population-based thresholds with intraindividual reference change values. In a retrospective real-world cohort, reference change value elevation was more strongly associated with clinical relapse and active MRI than population-based threshold elevation. The authors translated concordant and discordant biomarker patterns into a proposed monitoring algorithm linking them to responses such as repeat measurement, closer surveillance, earlier MRI and consideration of treatment modification when disease activity was corroborated (19). This approach illustrates how longitudinal biomarker interpretation might be operationalized, but the proposed algorithm was not prospectively evaluated. Its clinical plausibility should not be confused with evidence that following it improves decisions or patient outcomes.

The decisive evidence requires comparison of management strategies rather than biomarker concentrations, prognostic models or intended decisions. MultiSCRIPT-Cycle 1 represents an important step in this direction. This pragmatic, multicenter, 1:1 randomized trial embedded in the Swiss Multiple Sclerosis Cohort plans to include 915 patients with RRMS. It compares usual care with a strategy adding 6-monthly sNfL monitoring to clinical, disability and MRI assessment. In the intervention group, normalized sNfL percentiles and Z-scores are provided to clinicians, and prespecified algorithms support their integration into personalized treatment decisions. These algorithms inform rather than dictate management: whether and how the physician and patient act on the result remains their decision (7).

MultiSCRIPT is particularly relevant because its objective extends beyond determining whether sNfL changes management. It evaluates whether a care strategy incorporating serial sNfL monitoring improves patient-relevant outcomes compared with usual care. The co-primary outcomes assess disease activity and health-related quality of life, while secondary outcomes include relapses, disability worsening, MRI activity and clinical, patient-reported, safety, healthcare-utilization and economic outcomes (7). The intervention being tested is therefore not the biomarker itself, but a management strategy in which biomarker information is incorporated into shared clinical decision-making.

Together, these studies illustrate a progressive evidence chain from biomarker information to decision impact, operational implementation and patient benefit, as summarized in **Figure 4**. Current evidence has reached different levels along this pathway: van Lierop et al. provide evidence of decision impact; NeuroFilMS will examine real-world implementation and decision-making; Yaldizli et al. and de las Heras Flórez et al. propose ways of linking biomarker patterns to clinical responses; and MultiSCRIPT directly compares a biomarker-informed care strategy with usual care.

**Figure 4:**
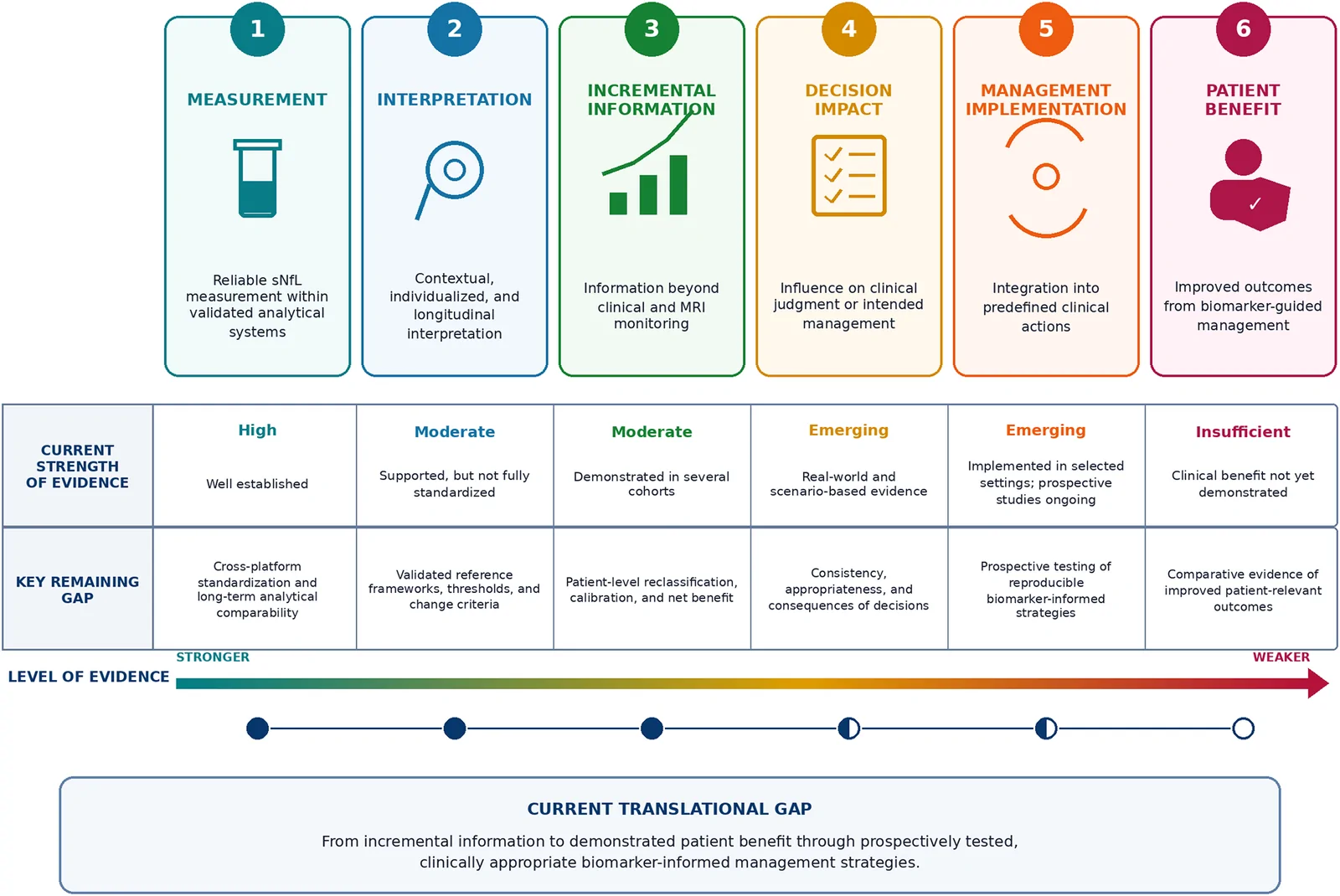
From sNfL information to improved outcomes: current evidence and remaining translational gaps. Current evidence is high for reliable measurement, moderate for individualized interpretation and incremental information, emerging for decision impact and management implementation, and insufficient for demonstrated patient benefit. The critical remaining gap is whether a predefined sNfL-informed management strategy improves patient-relevant outcomes compared with otherwise similar care. Unlike **Figure 1**, which presents the methodological requirements for clinical actionability, **Figure 4** summarizes the current position of the evidence along the translational pathway (2, 6–8, 14–16, 19, 46). Evidence-strength labels are qualitative judgments from the narrative synthesis and are not formal GRADE ratings. MRI, magnetic resonance imaging; sNfL, serum neurofilament light chain.

Importantly, the current absence of demonstrated outcome benefit should not be interpreted as an argument for restricting sNfL exclusively to research settings. Rather, monitored implementation within prospective cohorts, registries, and pragmatic study designs may itself provide a means of generating the decision-impact and outcome-level evidence that is currently lacking. Such approaches provide an intermediate pathway between unrestricted adoption and trial-only use, allowing biomarker-informed management to be evaluated under real-world or pragmatic conditions while preserving predefined protocols and outcome assessment (7, 8).

Future studies should follow this strategy-level logic. They should prespecify how an abnormal or changing sNfL signal is defined, when it should be considered clinically relevant, which actions should follow different biomarker patterns and against what comparator biomarker-informed care should be evaluated. They should also document whether the proposed action was followed and, when it was not, why the clinician and patient chose an alternative course.

Outcomes should extend beyond biomarker performance and conventional measures of disease activity. They should include potential harms, unnecessary investigations, treatment exposure, patient-reported outcomes, quality of life, healthcare utilization and cost-effectiveness. The evidentiary threshold should be particularly high when biomarker information contributes to consequential decisions such as treatment escalation, de-escalation or discontinuation.

Until this is demonstrated, current evidence supports biomarker-informed care rather than fully biomarker-guided care.

This review has limitations. Its narrative and purposive design was chosen to integrate heterogeneous evidence spanning analytical validation, prognostic modeling, clinical decision research, and implementation science, but it does not provide the exhaustive study identification, duplicate screening, or formal risk-of-bias assessment of a systematic review. Reliance on a single bibliographic database may have omitted relevant evidence, and selection and interpretation by a single author may have introduced selection or interpretative bias. The proposed actionability ladder is a conceptual framework rather than a validated qualification instrument, and the clinical responses summarized here remain decision-support propositions rather than tested treatment algorithms. Finally, rapid evolution of assays, reference resources, and ongoing prospective studies means that some conclusions—particularly those concerning thresholds, longitudinal metrics, and outcome benefit—will require updating as new evidence emerges.

## 9 Conclusions

sNfL has moved beyond being merely a promising biomarker of neuroaxonal injury in MS. The relevant question is no longer whether it is associated with inflammatory activity, treatment response or future outcomes, but whether it provides sufficiently reliable and incremental information to improve the assessment and management of an individual patient. Answering this question requires more than biomarker validation: it requires analytically comparable measurement, individualized and longitudinal interpretation, demonstration of added value beyond established monitoring and a proportionate clinical response to the information obtained.

Current evidence supports incorporating sNfL into clinical reasoning alongside clinical assessment and MRI. Its most plausible contribution is to modify residual uncertainty, particularly when an appropriately interpreted result is discordant with an otherwise reassuring assessment. However, elevated values are not specific for MS activity, and non-elevated values cannot override convincing clinical or MRI evidence or exclude all clinically relevant disease biology, particularly relapse-independent progression. These limitations are unlikely to be resolved by a universal threshold or by further refinement of sNfL alone and support the development of complementary biomarker and multimodal approaches.

The remaining translational challenge is to determine whether predefined strategies incorporating sNfL into clinical management improve patient-relevant outcomes compared with otherwise similar care. Until such benefit is demonstrated, sNfL should inform and refine clinical decisions, but should not independently determine them.
